# Phytochemical Analysis and Antimicrobial Activity of *Terminalia bellirica* (Gaertn.) Roxb. and *Terminalia chebula* Retz. Fruit Extracts Against Gastrointestinal Pathogens: Enhancing Antibiotic Efficacy

**DOI:** 10.3390/microorganisms12122664

**Published:** 2024-12-22

**Authors:** Gagan Tiwana, Ian Edwin Cock, Matthew James Cheesman

**Affiliations:** 1 School of Pharmacy and Medical Sciences, Gold Coast Campus, Griffith University, Gold Coast 4222, Australia; g.tiwana@griffith.edu.au; 2 School of Environment and Science, Nathan Campus, Griffith University, Brisbane 4111, Australia; i.cock@griffith.edu.au

**Keywords:** medicinal plants, plant-derived antimicrobials, gallotannins, gastrointestinal pathogens, herbal antidiarrheals, combinational therapies, LC-MS

## Abstract

*Terminalia bellirica* (Gaertn) Roxb. and *Terminalia chebula* Retz. are significant botanicals in ancient Ayurvedic medicine. They are renowned for their therapeutic properties, notably in addressing gastrointestinal (GI) diseases. These plants have undergone thorough examination related to their antibacterial, anti-inflammatory, and antioxidant properties, which make them highly efficient natural treatments for controlling gastrointestinal infections. The current research demonstrated the antibacterial efficacy of fruit extracts of *Terminalia bellirica* and *Terminalia chebula* against *Bacillus cereus*, *Shigella sonnei*, *Shigella flexneri*, and *Salmonella typhimurium*. We performed disc diffusion and liquid microdilution experiments to evaluate the antibacterial efficacy. All extracts of *Terminalia bellirica* and *Terminalia chebula* showed good antibacterial effects against *B. cereus* and *S. flexneri*. The minimum inhibitory concentration (MIC) values ranged from 94 µg/mL to 556 µg/mL. The methanolic extracts from both plants also showed noteworthy antibacterial activity against *S. sonnei* and *S. typhimurium*, with MIC values of 755 µg/mL for both. Fractional inhibitory concentration studies revealed additive interactions between some conventional antibiotics and the plant extracts when used concurrently. Liquid chromatography–mass spectrometry (LC-MS) analyses revealed that the *T. bellirica* and *T. chebula* extracts contained various tannins including methyl gallate, propyl gallate, gallic acid, and ellagic acid. Lethality assays conducted using *Artemia franciscana* Kellogg nauplii indicated that all the plant extracts are non-toxic. The antibacterial properties and absence of toxicity in *T. bellirica* and *T. chebula* fruit extracts indicate their potential for antibiotic development, warranting additional mechanistic and phytochemical studies.

## 1. Introduction

Diarrhoeal disease is the third most common cause of mortality among children aged five and below, causing more than 443,000 deaths annually [1]. Diarrhoea can persist for multiple days and deplete the body of vital water and minerals essential for survival. Historically, severe dehydration and fluid depletion were the primary factors leading to fatalities related to diarrhoea. Diarrhoea often arises as a manifestation of an infection in the gastrointestinal tract, which can be triggered by a range of bacterial, viral, and parasitic microorganisms [1].

Common bacterial pathogens involved in the development of diarrhoeal infections include *Escherichia coli*, *Salmonella* spp., *Shigella* spp., *Campylobacter* spp., *Clostridium difficile*, *Yersinia* spp., etc. [1]. Notably, gastrointestinal (GI) bacterial infections are increasingly contributing to the overall mortality associated with diarrhoea. The individuals most vulnerable to life-threatening diarrhoea include children and the elderly, people with malnutrition or weakened immune systems, as well as individuals living with HIV. The transmission of GI infections occurs through the consumption of contaminated food or drinking water, or person-to-person contact due to inadequate hygiene practices [1]. The global economic burden of diarrheal diseases is substantial, costing an estimated USD 12 billion annually in healthcare expenses, productivity losses, and long-term disability [2]. This significant financial impact underscores the need for improved prevention and treatment strategies.

Bacillary dysentery is a condition that is marked by severe diarrhoea, and *S. flexneri* and *S. sonnei* are two of the most important pathogens that are linked with it [3]. These pathogens are highly contagious, especially in areas with inadequate sanitation. Due to their vulnerability, these infections pose a substantial public health issue, particularly to the elderly and young children [3]. In addition, *S. typhimurium* is a prevalent cause of foodborne illness, spreading through contaminated poultry and eggs, resulting in symptoms ranging from mild gastroenteritis to more severe forms of the disease [4]. Furthermore, *B. cereus* produces toxins that can result in food poisoning, leading to vomiting and diarrhoea [5]. This condition is often linked to inadequate food storage or the reheating of starchy foods. Additionally, *Bacillus* species pose an additional challenge due to their psychotropic characteristics, allowing them to survive and grow at low temperatures. This reduces the effectiveness of refrigeration as a food preservation method, necessitating alternative strategies to address contamination and spoilage. *Bacillus cereus* infections generally resolve spontaneously, although they pose a considerable risk to vulnerable populations, including children and the elderly [5].

Multiple strains of *S. flexneri* and *S. sonnei* have recently developed increased resistance to a range of antibiotics including fluoroquinolones, cephalosporins, azithromycin, sulphonamides, tetracycline, and streptomycin [6,7]. Similarly, some *S. typhimurium* strains have developed resistance to ampicillin, chloramphenicol, streptomycin, sulphonamides, and tetracycline [8]. Additionally, some *B. cereus* strains exhibit significant resistance to β-lactam antibiotics including penicillin G and cefotaxime [9,10].

Traditional herbal treatments may be useful in developing novel therapies to combat antimicrobial-resistant gastrointestinal infections. Secondary metabolites of herbal plants, such as alkaloids, flavonoids, phenolic acids, tannins, and terpenoids play a crucial role in a plant’s defense system and protect from infections [11]. Ayurvedic medicinal plants are a promising target for developing novel antibiotic chemotherapy treatments due to their easy accessibility, cost-effectiveness, and expanding evidence-based efficacy [11]. A previous study from our group highlighted the role of traditionally used Ayurvedic medicinal plants in the treatment and management of dysentery, diarrhoea, and other gastrointestinal infections [12]. In particular, *Terminalia bellirica* (Gaertn.) Roxb. and *Terminalia chebula* Retz fruit decoctions, infusions, or pulp were highlighted for their traditional uses to treat dysentery and chronic diarrhoea. Numerous other medicinal plants, including *Croton lechleri* Mṻll. Arg., *Mentha piperita* L., *Camellia sinensis* (L.) Kuntze, *Emblica officinalis* Linn., *Phyllanthus niruri* L., and *Azadirachta indica* A. Juss. have also been reported to possess antidiarrhoeal activity [12,13]. Furthermore, several constituents of these plants, including apigenin, friedelin, 1,8-cineole, eriosematin E, and stachysrosane (1) and (2) have been found to exhibit antidiarrhoeal activity in a castor-oil-induced diarrhoea model [13].

Potent antibacterial activity has previously been reported for aqueous and ethanolic fruit extracts of *T. bellirica* and *T. chebula* against *S. flexneri* and *S. sonnei*, with MIC values in the 0.01 to 100 µg/mL range [14]. However, the methodology and preparation of the extracts were not clearly stated, making these results difficult to duplicate. In addition, no toxicity studies were conducted, and the antibacterial assays were not performed in duplicates or triplicates to confirm the results. In contrast, another study reported very low antibacterial activity for aqueous *T. bellirica* fruit extracts against *S. flexneri*, with an MIC value of 12,500 µg/mL [15]. Similarly, one study showed low antibacterial activity of aqueous and alcoholic *T. bellirica* fruit extracts in the agar well diffusion method against *S. typhimurium* (MIC = 12,500 µg/mL) [16]. Aqueous *T. bellirica* fruit extracts have also been reported to reduce bacterial loads in an *S. typhimurium*-infected animal model [17]. In a previous investigation from our group, we noted that *T. bellirica* and *T. chebula* fruit extracts (aqueous and methanolic) exhibit low-to-moderate antibacterial activity against *S. typhimurium* and *S. sonnei* [18]. In contrast, the methanolic fruit extracts of both species exhibited high bacterial growth–inhibitory activity against *S. flexneri*, with MIC values of 287 µg/mL and 276 µg/mL, respectively.

Our research study examined antibacterial activities and the phytochemical constituents of the methanolic, aqueous, and ethyl acetate extracts of *T. bellirica* and *T. chebula*. *Shigella flexneri, Shigella sonnei*, *Salmonella typhimurium*, and *Bacillus cereus* were used for the antibacterial analyses. Liquid chromatography–mass spectrometry (LC-MS) was conducted to identify phytochemicals within the *T. bellirica* and *T. chebula* extracts, with a focus on identifying noteworthy flavonoids, tannins, and terpenoid compounds. Additionally, combinational effects between any active plant extracts and selected reference antibiotics were investigated, alongside extract toxicity determinations using *Artemia franciscana* Kellogg nauplii (brine shrimp) lethality assays.

## 2. Materials and Methods

### 2.1. Sources of Plant Samples

The dried and powdered *Terminalia bellirica* fruit (manufacturer’s code: BNFP/01) used in this study was manufactured by Organic Prime and supplied by Navafresh, Australia. The *Terminalia chebula* fruit powder (manufacturer’s code: HRP1020) was formulated by Aarshaveda (Sattvic, Melbourne, Australia). The supplier’s website listed the plant materials under their traditional Ayurvedic names of Bhitaki and Baheda (for *T. bellirica*) and Haritaki and Harad (for *T. chebula*). The quality and authenticity of the plant materials were confirmed by the supplier. Voucher specimens of the *T. bellirica* fruit (NBG-TB0220GU) and *T. chebula* fruit (NBG-TC0220GU) are stored at the School of Pharmacy and Medical Sciences on the Gold Coast campus of Griffith University.

### 2.2. Preparation of Extracts

Individual masses (1 g) of either *T. bellirica* or *T. chebula* fruit powders were weighed into six 50 mL Falcon tubes. Autoclave sterilised deionised water (AQ), as well as methanol (MeOH) or ethyl acetate (EtOAc) were then added separately to individual tubes to make a total volume of 50 mL [18,19]. The MeOH and EtOAc solvents were AR grade and were supplied by ChemSupply (Gillman, Australia). The extraction tubes were allowed to extract at ambient temperature for 24 h using continuous rolling (30 rpm). Subsequently, the samples were filtered using Whatman No. 54 filter paper (Sigma-Aldrich, Melbourne, Australia) into 50 mL Falcon tubes under vacuum. AQ samples were subjected to lyophilisation using an Alpha 1–4 LSC plus benchtop freeze dryer (Martin Christ, Osterode am Harz, Germany) for three days to freeze-dry. The organic solvent extracts were evaporated at 40 °C for two days, or until evaporation was completed (assessed as a constant mass with repeated weighing). To determine the final yields, the weight of all dried extracts was measured. The extracts were then reconstituted in 10 mL of a 1% solution of dimethyl sulfoxide (DMSO; Merck, Macquarie Park, Australia) and subjected to three rounds of sonication. Each round consisted of 20-s pulses from a probe sonicator set at 1 kHz, with a 30-s break between pulses. The extracts were sterilised by filtration through 0.22 µm filters (Sarstedt, Mawson Lakes, Australia) and then stored at −20 °C until they were used.

### 2.3. Antibiotics and Bacterial Strains

Amoxycillin (potency = 900 µg/mg), chloramphenicol (≥98% HPLC purity), ciprofloxacin (HPLC purity ≥98%), erythromycin (potency ≥850 µg/mg), gentamicin (HPLC purity ≥98%), oxacillin (TLC purity ≥95%), penicillin G (potency = 1440–1680 µg/mg), polymyxin B (HPLC purity ≥90%), tetracycline (HPLC purity ≥95%), and vancomycin (potency ≥900 μg/mg) were purchased from Sigma-Aldrich (Melbourne, Australia) and included in this study as antibiotic controls. Stock solutions (1 mg/mL) of all antibiotics were prepared for use in the liquid microdilution experiments and stored at a temperature of −20 °C until they were needed. Preloaded standard Augmentin^®^ (15 µg), cefoxitin (30 µg), chloramphenicol (30 µg), ciprofloxacin (1 µg), erythromycin (10 µg), gentamicin (10 µg), oxacillin (1 µg), penicillin G (10 IU), polymyxin B (300 IU), tetracycline (30 µg), and vancomycin (30 µg) antibiotic susceptibility testing discs were purchased from Oxoid Ltd. (Thebarton, Australia). Additionally, 10 µL of an amoxycillin solution (0.01 mg/mL) was loaded by infusion into sterile filter paper discs and used as an additional antibiotic control. Broth microdilution experiments were performed using liquid solutions of all reference antibiotics, except for Augmentin^®^ and cefoxitin, which were unavailable.

Bacterial species were purchased from the American Type Culture Collection (ATCC; Manassas, VA, USA) and included *Shigella sonnei* (ATCC 25931), *Salmonella typhimurium* (*Salmonella enterica* serovar *Typhimurium*; ATCC 14028), *Shigella flexneri* (ATCC 12022), and *Bacillus cereus* (ATCC 14579). Oxoid Ltd. (Thebarton, Australia) supplied the Mueller–Hinton (MH) agar and broth (Oxoid Ltd., Australia) to sustain bacterial growth. Each MH agar plate was prepared to 4 mm agar depth according to the manufacturer’s instructions.

### 2.4. Evaluation of Bacterial Susceptibility

The susceptibility of the bacterial test panel to the plant extracts and control antibiotics in an MH agar model system was examined using standard disc diffusion methods [19]. Briefly, colonies were isolated from streaked agar plates and subsequently used to inoculate 40 mL of fresh MH broth. The bacteria were cultivated at 37 °C for 18–24 h. McFarland 0.5 standards were prepared for each strain using the individual bacterial cultures. The 0.5 McFarland standard cultures (100 µL) were spread evenly onto the surface of MH agar plates. Sterile 6 mm diameter filter paper discs were carefully placed onto the MH agar surface to avoid disturbing the spread bacterial surface. The extracts (10 µL dissolved in 1% DMSO) were subsequently added individually to the discs. Additionally, reference antibiotic discs containing the control antibiotics were included on all plates. All plates were incubated at 37 °C until confluent growth was achieved (18–24 h). The samples (extracts and reference antibiotics) were analysed in at least triplicate. The zones of inhibition (ZOI) surrounding all filter discs were measured to assess bacterial susceptibility to the extracts and controls. Samples lacking antibacterial activity were deemed to have a ZOI of 6 mm (equal to the diameter of the test disc).

### 2.5. Minimum Inhibitory and Fractional Inhibitory Concentrations

The extracts and most of the reference antibiotics were subjected to broth microdilution assays in order to obtain minimum inhibitory concentration (MIC) values, as described previously [18,19]. All samples and controls were tested in two independent experiments, each with internal duplicates (*n* = 4). Values ≥10,000 μg/mL were categorised [19] as inactive; those falling within the range of 2000 to 10,000 μg/mL were deemed to possess low activity; MIC values ranging from 1000 to 2000 μg/mL were categorised as moderate activity; values between 400 and 1000 μg/mL were considered noteworthy activity; and values between 100 and 400 μg/mL were classified as good activity. Any MIC values less than 100 μg/mL were considered to have a high level of activity.

Plant extracts and reference antibiotics that showed antibacterial activity with MIC ≤ 3000 µg/mL and ≤2.5 µg/mL, respectively, were combined in equal ratios (50:50) and retested using the MIC methodology described above. The sum of the fractional inhibitory concentrations (ΣFIC) for each combination was determined using the equations shown below (a = extracts; b = antibiotics):

*FIC*(*a*) = *MIC* (*a in combination with b*)/*MIC* (*a independently*)

*FIC*(*b*) = *MIC* (*b in combination with a*)/*MIC* (*b independently*)

The sum of the fractional inhibitory concentrations (∑FIC) was determined by the following:

∑*FIC* = *FIC*(*a*) + *FIC*(*b*)

The classifications of the interactions included synergistic (∑FIC ≤ 0.5); additive (∑FIC > 0.5–≤ 1.0); indifferent (∑FIC > 1.0–≤ 4.0); or antagonistic (∑FIC > 4.0) [20].

### 2.6. Toxicity Assays

Extracts and control samples were subjected to *Artemia franciscana* Kellogg nauplii lethality assays (ALA) [21] as an indicator of their toxicities. For testing, the extracts were diluted in artificial seawater (Red Sea Pty. Ltd., Springfield, Australia; 32 g/L) to a concentration of 2 mg/mL. Then, 400 µL of each test sample (or 400 µL of the artificial seawater negative control) were added to sterile 48-well plates followed by the addition of approximately 50 newly hatched *A. franciscana* nauplii (the brine shrimp eggs were sourced from Aquabuy, Auburn, Australia). Negative controls (400 µL of artificial seawater) and sodium azide positive controls (10 mg/mL) were included in triplicate on all plates for comparison. The plates were incubated for 24 h at 25 °C ± 1 °C. Following this incubation, the viability of the nauplii was assessed visually. LC_50_ values (the concentration of extract or control needed to cause the death of 50% of *A. franciscana* nauplii) were determined by graphically plotting the mean percentage of mortality from three separate experiments and determining the concentration that caused 50% mortality using Probit analysis.

### 2.7. Metabolomics LC-MS Analysis of the Extracts

A Vanquish ultra-high-performance liquid chromatography (UHPLC) system (Thermo Fisher Scientific in Waltham, MA, USA) was used to separate and identify phytochemical extract components by non-targeted headspace metabolic profiling. The metabolomic profiling analysis utilised chromatographic parameters previously developed by our group [19]. Briefly, individual phytochemical components were separated using an Accucore ™ RP-MS column (100 mm × 2.1 mm), with a particle size of 2.6 μm, connected to an Orbitrap Exploris 120 mass spectrometer (Thermo Fisher Scientific). The injection concentration of plant samples was 1 mg/mL, and the chromatography system was run at a flow rate of 0.6 mL/min. The following elution solvents and gradient were used to elute the compounds from the column: (A) 0.1% volume/volume (*v*/*v*) formic acid dissolved in ultrapure water; and (B) acetonitrile (MeCN) containing 0.1% *v*/*v* formic acid, 5% solvent B for 5 min, then, there was a gradual increase of 5% to 30% of solvent B over 5 min, followed by a 3-min isocratic step at 30% solvent B. Next, there was a linear gradient from 30% to 90% of solvent B over 4 min. An isocratic elution step at 90% of solvent B occurred for 4 min, which was then used to clean the column of any non-polar compounds.

The mass spectral analyses of all compounds were analysed as they eluted using an Orbitrap Exploris 120 mass spectrometer in the information-dependent acquisition (IDA) mode. The mass spectral analysis used electrospray ionisation (ESI) in negative ionisation mode (ranging from 2.5 kV to 5 kV). The vaporiser was maintained at a temperature of 350 °C. Compound identification used Compound Discoverer^TM^ 3.3. Background absorbance (determined by the blank file) was subtracted from each test chromatogram to make a list of identified extract compounds. Utilising Mz Cloud, ChemSpider, Predicted Compositions, and MassList, Compound Discoverer correlated mass signals with established compounds. Compound Discoverer 3.3 generated Excel files for each extract. The files were subsequently examined to identify potential speculative compounds. The relative abundance of the identified compounds was determined as a percentage of the total area of the chromatogram.

### 2.8. Statistical Analyses

Three independent experiments were conducted to generate the ZOI results to generate means ± standard error of the mean (±SEM). Using one-way analysis of variance (ANOVA), the statistical significance between the treatment and negative control groups was investigated; *p*-values <0.05 were considered statistically significant, and *p*-values < 0.01 were considered statistically highly significant.

## 3. Results

### 3.1. Antibacterial Assays

The extract concentrations were comparable for both *Terminalia* species. The highest yields were consistently achieved for the MeOH and AQ extractions. In contrast, EtOAc extraction produced significantly lower yields for both plant species. The extract yields of *T. bellirica* AQ, MeOH, and EtOAc were 50.2, 48.3, and 3.6 mg/mL, respectively. In contrast, *T. chebula* yielded 35.6, 48.3, and 4.9 mg/mL for the same solvents.

**Figure 1:**
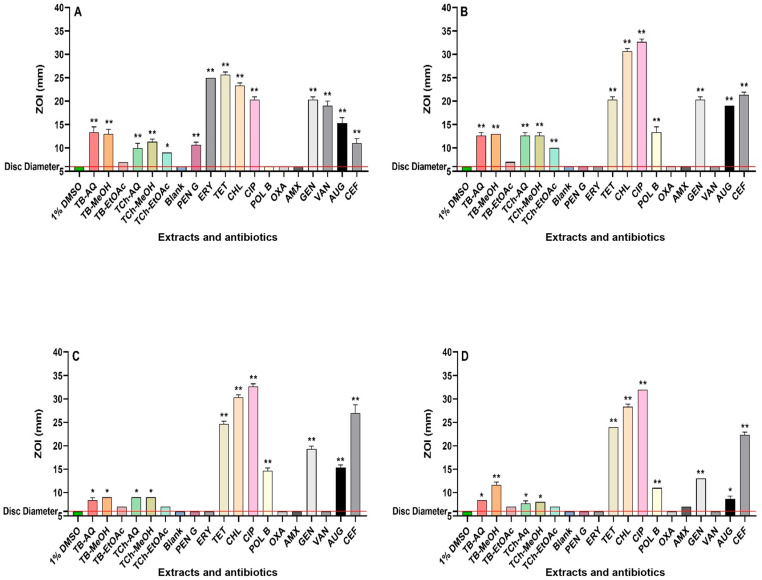
Antimicrobial activity of *T. bellirica* and *T. chebula* fruit extracts in the disc diffusion assays against (**A**) *B. cereus*, (**B**) *S. flexneri*, (**C**) *S. sonnei*, (**D**) *S. typhimurium*. TB-AQ = *T. bellirica* aqueous (50.2 mg/mL); TB-MeOH = *T. bellirica* methanolic (48.3 mg/mL); TB-EtOAc = *T. bellirica* ethyl acetate (4.9 mg/mL). TCh-AQ = *T. chebula* aqueous (35.6 mg/mL); TCh-MeOH = *T. chebula* methanol (48.3 mg/mL); TCh-EtOAc = *T. chebula* ethyl acetate (4.9 mg/mL). Negative controls = 1% DMSO and blank = sterile water. Reference antibiotics: PEN G = penicillin G (10 IU), ERY = erythromycin (10 µg), TET = tetracycline (30 µg), CHL = chloramphenicol (30 µg), CIP = ciprofloxacin (1 µg), POL B = polymyxin B (300IU), OXA = oxacillin (1 µg), AMX = amoxycillin (10 µL of 0.01 mg/mL stock solution), GEN = gentamicin (10 µg), VAN = vancomycin (30 µg), AUG = Augmentin^®^ (15 µg), CEF = cefoxitin (30 µg). Horizontal red lines on the *y*-axis at 6 mm indicate the disc diameter used in the assay. Mean values (±SEM) are reported from three independent studies. *p*-values < 0.05 are represented with a single asterisk symbol (*), while *p*-values < 0.01 are represented with a double asterisk symbol (**).

All extracts (and control antibiotics) were then subjected to solid-phase disc diffusion assays (Figure 1) as well as broth microdilution assays (Table 1). These assays revealed that *S. flexneri*, *S. sonnei*, and *S. typhimurium* were resistant to penicillin G, oxacillin, amoxycillin, and erythromycin. The extracts of both plants demonstrated similar results against *B. cereus* in agar disc diffusion assays, exhibiting ZOIs ranging from 9 to 13 mm. However, the EtOAc *T. bellirica* extract was inactive on agar against that bacterium. The *T. bellirica* AQs, MeOH, and EtOAc extracts produced MIC values of 392, 94, and 450 µg/mL, respectively, against *B. cereus*. In contrast, MIC values of 556, 377, and 306 µg/mL were determined for the *T. chebula* AQ, MeOH, and EtOAc extracts, respectively, against *B. cereus*.

All of the *T. bellirica* (except for EtOAc) and *T. chebula* extracts displayed substantial antibacterial activity against *S. flexneri* in the agar diffusion assays, with ZOIs ranging from 10 to 13 mm. This antibacterial activity is promising and comparable to the reference antibiotics such as polymyxin B. In broth microdilution assays, all extracts from both plant species demonstrated significant growth inhibition activity against *S. flexneri* (MIC values between 278 and 450 µg/mL).

In the disc diffusion assays, only the AQ and MeOH extracts of each plant demonstrated antibacterial activity against *S. sonnei*, with ZOIs of 8–9 mm. The *T. bellirica* AQ and MeOH extracts demonstrated antibacterial activity against *S. sonnei*, with MIC values of 1569 and 755 µg/mL, respectively. In contrast, all the *T. chebula* extracts displayed only low-to-moderate antibacterial activity against *S. sonnei* in broth dilution assays (MICs ranging from 1225 to 2225 µg/mL).

The AQ and MeOH extracts of *T. bellirica* and *T. chebula* displayed antibacterial activity against *S. typhimurium* in the disc diffusion assay, with ZOIs ranging from 7.5 to 11.5 mm. Only the methanolic extract of *T. bellirica* exhibited a statistically significant ZOI (11.5 mm), comparable to polymyxin B, gentamicin, and Augmentin. The broth microdilution assays revealed low antibacterial activities of *T. bellirica* (AQ) and *T. chebula* (AQ and MeOH), with MIC values in the range of 2225–3138 µg/mL. However, the MeOH *T. bellirica* extract showed noteworthy antibacterial activity against *S. typhimurium* (MIC = 755 µg/mL).

### 3.2. Combinational Studies: ƩFIC Determinations

For antibiotics and extracts that displayed substantial antibacterial activity when tested alone, combinations were also evaluated to identify interactive effects between the two components against the bacterial strains tested in this study (Table 2). Only extracts and the antibiotic components that demonstrated substantial antibiotic efficacy against the bacterial species under investigation were tested in combination as MIC values are required for both components in the combination to calculate the ƩFIC values. No synergistic interactions were detected in any of the combinations analysed. Thirty-five combinations resulted in additive effects, whilst seventy-nine combinations exhibited non-interaction. Notably, seventeen combinations exhibited antagonistic effects, particularly in combinations containing polymyxin B as the antibiotic component.

### 3.3. LC-MS Metabolomics Profiling and Compound Identification

The metabolomic profiles of all extracts were evaluated using LC-MS profiling analysis, and (where possible) compounds were identified by comparison to multiple databases. Whilst our study aimed to identify a wide variety of compounds, the flavonoid, tannin, and terpenoid compounds were emphasised. The majority of the extract compounds were eluted during the 30% and 90% acetonitrile gradient elution phase of the chromatogram [22], indicating that most extract components exhibited relatively high polarity [23]. In contrast, the organic acid and amine components exhibited an enhanced tendency to elute in polar environments, resulting in their early appearance in the chromatogram. The low-polarity lipophilic compounds, which demonstrate a greater affinity for the non-polar stationary phase, eluted later as the gradient continued, at high ACN concentrations. To construct a prospective inventory of all the discovered phytochemicals, only those compounds that fully matched with reported data in at least one of the screened databases were selected [22]. In this investigation, we have concentrated on the phenolic acid, tannin, flavonoid, and terpenoid compounds (Table 3). These compounds are important because of their various biological functions. They have antioxidant, anti-inflammatory, and antibacterial characteristics, making them useful in health, medicine, and nutrition. Their activities in plant defence also provide natural sources for drug research and therapeutic uses, which are beneficial to human health and disease prevention.

### 3.4. Toxicity Quantification

Plant extract toxicity was assessed by *Artemia franciscana* lethality assays (ALA). The extracts were categorised as toxic if they produced LC_50_ values ≤ 1000 µg/mL after a 24-hour exposure period [18]. The findings for all of the extracts were similar to those of the artificial seawater negative control (*p* > 0.05) since they failed to induce ≥50% mortality at 1000 µg/mL and were thus deemed non-toxic.

## 4. Discussion

Our study reports significant growth inhibition activity for the AQ and MeOH *T. bellirica* and *T. chebula* fruit extracts against a panel of four gastrointestinal bacterial pathogens. With some notable exceptions, the EtOAc extracts generally displayed less potent antibacterial activity. The variations in extraction yields and the concentrations of the different extracts may account for these differences. MeOH and AQ are substantially higher in polarity than EtOAc, allowing for the extraction of greater quantities of mid-to-high polarity phytochemicals. In contrast, EtOAc mainly extracts mid-to-low polarity compounds, resulting in substantially less complexity and lower compound yields [24]. Additionally, lower polarity or larger-size compounds generally have reduced diffusion rates through solid-phase agar, leading to a diminished apparent antibacterial effectiveness in disc diffusion assays [25]. The depth and uniformity of agar in petri dishes can also influence the size of zones of inhibition (ZOIs) in agar diffusion experiments [26]. The solubility of these phytochemicals in broth is influenced by their polarity [27], which affects their dissolution and may result in inaccurate MIC values. Whilst our study highlights the antibacterial potential of *T. bellirica* and *T. chebula* extracts against the four bacterial pathogens screened, future research must broaden the scope of bacterial strains tested to assess the efficacy of plant extracts on additional antibiotic-resistant strains. The specific phytochemical profiles of the extracts may have profound effects on the antibacterial potency and/or specificity of the extracts. Flavonoids, tannins, and terpenoids exert antibacterial activity via a variety of mechanisms, including disrupting bacterial cell walls and membranes, altering internal pH levels, inhibiting enzymatic activity, and interfering with DNA replication [12]. Furthermore, the combined effect of these compounds improves their overall antibacterial effectiveness compared to the individual components in isolation. Plant extracts may also inhibit toxin production and quorum sensing systems, thereby further diminishing the virulence of the bacterial pathogens and their capacity to form biofilms [6,28,29,30,31].

All reference antibiotics (except polymyxin B) showed varying levels of antibacterial activity against *B. cereus*. Polymyxin B was ineffective against *B. cereus* as it specifically targets Gram-negative bacteria by binding to lipopolysaccharides in their outer membrane [32], which are absent in Gram-positive bacteria such as *B. cereus*. This bacterium possesses a thick peptidoglycan layer and lacks the requisite binding sites [33], which renders polymyxin B ineffective and results in intrinsic resistance. The reference antibiotics tetracycline, chloramphenicol, ciprofloxacin, polymyxin B, and gentamicin exhibited varying levels of antibacterial activity against *S. flexneri*, *S. sonnei*, and *S. typhimurium*. The ineffectiveness of penicillin G, erythromycin, oxacillin, amoxycillin, and vancomycin against *S. flexneri*, *S. sonnei*, and *S. typhimurium* can be attributed to multiple factors. These bacteria are Gram-negative and have an outer membrane that serves as a barrier [30,31], inhibiting the penetration of various antibiotics, including penicillin G and vancomycin, which primarily target Gram-positive bacteria. *Shigella* and *Salmonella* species frequently produce β-lactamase enzymes [34,35], which degrade β-lactam antibiotics including penicillin G, oxacillin, and amoxycillin, thereby rendering those antibiotics ineffective. The presence of efflux pumps and genetic mutations in these bacterial pathogens may further diminish the effectiveness of conventional antibiotics [36,37]. Additionally, vancomycin targets the peptidoglycan layer [38], which is not accessible in Gram-negative bacteria, thereby restricting its efficacy. The presence of these combined resistance mechanisms requires the implementation of alternative treatments capable of surmounting these challenges.

Our study also investigated into the application of the *T. bellirica* and *T. chebula* extracts in conjunction with conventional antibiotics. Combinational therapies have considerable therapeutic potential as many bacteria have evolved resistance to traditional antibiotics. The use of phytochemicals may provide novel strategies to inhibit or block these resistance mechanisms [39]. By combining antibiotics with plant extracts, we hoped to improve their efficiency and to possibly overcome the bacterial-resistance mechanisms. For example, Augmentin^®^ is a clinically used antibiotic chemotherapy that contains a combination of amoxycillin and clavulanic acid [40]. The clavulanic acid component of the combination inhibits β-lactamase enzymes by irreversibly attaching to their active site, thereby preventing antibiotic degradation. This allows the amoxycillin component of the Augmentin^®^ combination therapy to function with greater potency, even in bacteria otherwise resistant to its effects.

Interesting effects were seen when the extracts were combined with β-lactam antibiotics. Specifically, combinations containing any *T. chebula* extract, or AQ *T. bellirica* extract with penicillin G, oxacillin, or amoxycillin produced additive effects against *B. cereus*. Whilst our study did not determine the mechanism of potentiation, specific extract phytochemicals may inhibit the bacterial β-lactamase enzyme(s) via a mechanism similar to that of clavulanic acid [41,42]. Such an interaction would safeguard β-lactam antibiotics from degradation by bacterial enzymes, thereby enhancing their efficacy against bacterial pathogens that are otherwise resistant to their effects. Similarly, AQ *T. bellirica* extracts showed additive effects against *B. cereus* in combination with vancomycin. Vancomycin inhibits peptidoglycan synthesis, thereby targeting the bacterial cell wall in Gram-positive bacteria. The flavonoids, tannins, and phenolic acid components of that extract may directly target bacterial structures, disrupt membrane permeability, or inhibit bacterial enzymes associated with resistance, although these potentiation mechanisms remain to be confirmed [42]. Furthermore, the potential for additional antibiotic resistance development may be diminished by the diverse range of phytochemicals found in plant extracts, which may function by targeting different aspects of bacterial cell growth and survival.

Combinations of the extracts with several other classes of antibiotics also potentiated the antibacterial activity. Indeed, combining tetracycline with several *Terminalia* spp. extracts produced additive effects against all of the gastrointestinal pathogens screened in our study. Bacterial resistance to tetracycline is primarily associated with tetracycline-specific efflux pumps [43]. Thus, the *T. bellirica* and *T. chebula* extracts may have impaired the activity of tetracycline-specific efflux pumps, which would facilitate prolonged retention of tetracycline within bacterial cells, resulting in higher intracellular antibiotic concentrations, thereby enhancing the efficacy of tetracycline. Alterations in ribosomal structure may also contribute to tetracycline resistance; however, this pathway is substantially less prevalent [43]. Similarly, the *T. chebula* and *T. bellirica* extracts potentiated the effects of chloramphenicol against *B. cereus* and *S. sonnei*. Chloramphenicol exerts its antibiotic effects via blocking bacterial protein synthesis. It binds to the 50S ribosomal subunit in the bacterial ribosome, specifically interacting with the 23S rRNA component [44]. This binding inhibits peptidyl transferase activity, an essential mechanism for peptide bond formation in protein synthesis. The plant extracts may also influence additional mechanisms related to bacterial resistance, including the disruption of efflux pumps and modification of cell wall structures [42], potentially increasing bacterial susceptibility to chloramphenicol. Additional studies are necessary to identify which mechanism(s) is/are influenced by the *Terminalia* spp. extracts. Additionally, combining *T. bellirica* or *T. chebula* AQ and MeOH extracts with gentamicin produced additive effects against multiple bacterial pathogens. These extracts may include compounds that hinder bacterial resistance mechanisms, or alternatively, that interact with alternative targets within the bacteria, thereby enhancing gentamicin’s mechanism of action (disruption of protein synthesis), although this is yet to be evaluated [45].

Several antagonistic effects were also noted in our study. Identification of combinations that produce antagonism is important as it indicates extracts and antibiotic combinations that should be avoided. In particular, several combinations containing polymyxin B were antagonistic in *S. flexneri* and *S. sonnei*. Polymyxin B functions by disrupting bacterial cell membranes by binding to lipopolysaccharides, although it is pH sensitive, with a significantly lower efficacy in acidic or alkaline environments [46]. Combining plant extracts (which contain relatively high levels of organic acids) with polymyxin B may alter the pH of the broth, thereby impacting the efficacy of the antibiotic component, and reducing the total antibacterial activity. Alterations in pH may also influence bacterial physiology, thereby diminishing bacterial susceptibility to the combined effects of the polymyxin B and the plant extracts. Alternatively, the antagonistic interactions between the extracts and polymyxin B may be attributed to the binding of bioactive phytochemicals to polymyxin B, which may hinder its absorption into the target bacterial cells [47].

**Figure 2:**
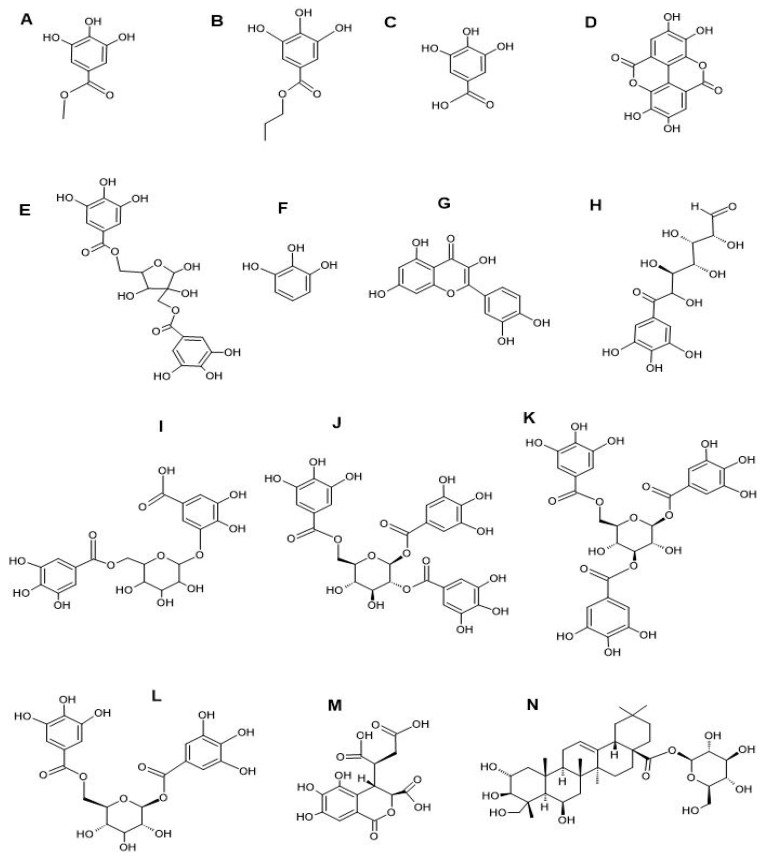
Structures of noteworthy compounds identified in the fruit extracts of *T. bellirica* and *T. chebula*. Methyl gallate (**A**), propyl gallate (**B**), gallic acid (**C**), ellagic acid (**D**), hamamelitannin (**E**), pyrogallol (**F**), quercetin (**G**), 6-galloylglucose (**H**), gallic acid 3-O-(6-galloylglucoside) (**I**), 1,2,6-trigalloyl-β-D-glucopyranose (**J**), 1,3,6-tri-O-galloyl-β-D-glucose (**K**), 1,6-bis-O-(3,4,5-trihydroxybenzoyl) hexopyranose (**L**), chebulic acid (**M**), and chebuloside II (**N**). Structures were prepared using Chemsketch Version 2024.

LC-MS metabolomics analysis of the *T. bellirica* and *T. chebula* fruit extracts revealed the presence of multiple tannins, flavonoids, terpenoids, and polyphenolic acid compounds (Table 3). The full lists of phytochemicals found detected in the individual plant extracts are provided in the supplementary files of our previous study [22]. Some important phytochemicals identified in both plant extracts include methyl gallate (Figure 2A), propyl gallate (Figure 2B), gallic acid (Figure 2C), ellagic acid (Figure 2D), hamamelitannin (Figure 2E), pyrogallol (Figure 2F), quercetin (Figure 2G), 6-galloylglucose (Figure 2H), gallic acid 3-O-(6-galloylglucoside) (Figure 2I), 1,2,6-trigalloyl-β-D-glucopyranose (Figure 2J), 1,3,6-tri-O-galloyl-β-D-glucose (Figure 2K), 1,6-bis-O-(3,4,5-trihydroxybenzoyl) hexopyranose (Figure 2L), chebulic acid (Figure 2M), and chebuloside II (Figure 2N). Notably, many of the tannin and flavonoid compounds identified in our study have also previously been identified in similar *T. bellirica* and *T. chebula* fruit extracts [48,49,50]. Additionally, previous studies using GC-MS headspace analysis also identified the volatile terpenoids endo-borneol, linalool, terpinene-4-ol, methoxy citronellal, pinocarveol, eucalyptol, carvone, camphor, L-fenchone, hyscylene, patchoulane, p-cumic aldehyde, and phenylbutanal in similar *T. bellirica* and *T. chebula* AQ, MeOH, and EtOAc fruit extracts, and those compounds may also contribute to the antibacterial and/or potentiation activities of those extracts [18].

Interestingly, studies have reported that combinations of methyl gallate (30 µg/mL) and marbofloxacin (0.015 µg/mL) inhibit adhesion and invasion of *S. typhimurium* in macrophage cell lines (RAW 264.7 cells) by 70% and 67%, respectively [51]. Moreover, this combination downregulated the *sdiA*, *srgE*, and *rck* quorum sensing genes of *S. typhimurium* by 53%, 62%, and 22%, respectively. Methyl gallate has been isolated and identified from the fruit extracts of *T. chebula* and has been reported to have antibacterial activity against *S. flexneri* and *S. sonnei*, with MIC values from 128 to 256 µg/mL, respectively [52]. That study also reported bacterial-membrane-damaging activity for methyl gallate, which was determined by membrane perturbation assays and transmission electron microscopy (TEM). Furthermore, previous studies have also reported that high levels of tetracycline and methyl gallate are accumulated in HeLa cells infected with *Shigella dysenteriae* [52]. This suggests that methyl gallate may be inhibiting tetracycline efflux pumps in *S. dysenteriae*. However, the combinational interactions of reference antibiotics and methyl gallate were not investigated in that study. Additionally, methyl gallate has weak β-lactamase inhibition activity compared to that of clavulanic acid [53]. This suggests that methyl-gallate-related antibacterial activity may result from cell membrane disruption, the inhibition of DNA synthesis, or targeting metabolic pathways. Indeed, methyl gallate has been reported to have substantial antibacterial activity against clinical isolates of *S. typhimurium*, with MIC values between 3.9 and 15.6 µg/mL [54]. That study also demonstrated that methyl gallate, when used in conjunction with ATPase inhibitors such as sodium azide (NaN_3_) and dicyclohexylcarbodiimide (DCCD), reduced *S. typhimurium* by approximately 40%. Furthermore, oral administration of methyl gallate significantly reduces *S. typhimurium* infections in BALB/c mice, with no observed clinical symptoms [54]. Another study also reported that methyl gallate (125 µg/mL) reduced the MIC of nalidixic acid from 500 µg/mL to 31.25 µg/mL against a nalidixic-acid-resistant *S. typhimurium* pathogenic strain, indicating that methyl gallate may negate the bacteria’s resistance mechanisms towards nalidixic acid [55].

Kang et al. (2018) demonstrated the notable inhibitory effect of gallic acid on *S. flexneri* biofilm formation through the regulation of the *mdoH* gene and the OpgH protein [56]. Gallic acid exhibits weak antibacterial activity against *S. flexneri*, with an MIC of 2000 µg/mL; however, that study did not examine the combination of gallic acid with conventional antibiotics. Another study reported antibacterial activities of gallic acid and hamamelitannin against multidrug-resistant strains of *S. typhimurium*, with MIC values of 256 µg/mL and 512–1024 µg/mL, respectively [57]. Fractional inhibitory combinational experiments demonstrated the additive effects of gallic acid with ampicillin, ceftiofur, and thiamphenicol, whilst hamamelitannin showed additive interactions with ceftiofur, cefotaxime, and marbofloxacin against *S. typhimurium* (ATCC14028). Additionally, the vitality of *S. typhimurium* biofilm formation was entirely suppressed by the combination of gallic acid and ceftiofur. Another study reported good antibacterial activity for ellagic acid against a panel of *Salmonella* species, including *S. paratyphi*, *S. choleraesuis* subsp., and *S. enteridis*, with MIC values of 20, 10, and 15 µg/mL, respectively [58]. A further study reported antibacterial efficacy for 3,3,4-tri-*O*-methylellagic acid and 3,4-di-*O*-methylellagic acid against *S. flexneri*, each with MICs of 5 µg/mL [59]. Both compounds also demonstrated antibacterial activity against *B. cereus*, with MIC values of 39 and 19 µg/mL, respectively. Notably, studies are deficient regarding the combinational interactions between ellagic acid and conventional antibiotics, and substantially more work is required. The synergistic effect of pyrogallol in conjunction with marbofloxacin against *S. typhimurium* has also been reported [60]. Pyrogallol exhibited MIC values of 128 µg/mL for all *S. typhimurium* isolates tested. The gentamicin protection assay demonstrated that pyrogallol (30 μg/mL), both independently and in conjunction with sub-MIC concentrations of marbofloxacin (0.015 μg/mL for susceptible strains and 0.25 μg/mL for marbofloxacin-resistant strains), inhibited 73% and 76% of the invading bacteria in Caco-2 cells, respectively [61]. Fluoroquinolone accumulation assays indicated that pyrogallol (at 30 μg/mL and 100 μg/mL concentrations) enhanced ciprofloxacin accumulation within *S. typhimurium* by 15% and 35%, respectively [60].

Our study identified several galloyl glucose derivatives within the *T. bellirica* and *T. chebula* extracts, including 6-galloylglucose, gallic acid 3-O-(6-galloylglucoside), 1,2,6-trigalloyl-β-D-glucopyranose, 1,3,6-tri-*O*-galloyl-β-D-glucose, and 1,6-bis-*O*-(3,4,5-trihydroxybenzoyl) hexopyranose. The presence of these compounds in the plant extracts may contribute to the noteworthy antibacterial activity of these extracts against the bacterial pathogens examined herein. A previous study demonstrated the antibacterial efficacy of 1,2,3,4,6-penta-*O*-galloyl-β-D-glucose against *B. cereus* and *S. typhimurium*, reporting MIC values of 32 µg/mL and 128 µg/mL, respectively [61]. However, studies examining the combinatorial interactions of galloyl glucose derivatives with conventional antibiotics are largely lacking.

ALA toxicity bioassays indicated that all *T. bellirica* and *T. chebula* extracts are non-toxic, supporting their safety for use as antibacterial agents; however, additional research with a panel of relevant mammalian cell lines should be performed in future studies to comprehensively evaluate whether these extracts are safe for medicinal use. Taken together, our findings suggest that *T. bellirica* and *T. chebula* fruit extracts could be an important source of antimicrobial compounds for future research into antibiotic development in the fight against bacterial gastrointestinal infections.

## 5. Conclusions

Traditionally, *T. bellirica* and *T. chebula* fruit extracts have shown promising results in the treatment of gastrointestinal infections because of their high concentrations of bioactive flavonoids, tannins, polyphenols, and terpenoids. Many of these phytochemicals have antibacterial, anti-inflammatory, and antioxidant activities that can reduce pathogenic microorganisms and improve gut health. Plant-based antimicrobials provide a natural, sustainable alternative to conventional antibiotics, lowering the danger of antibiotic resistance. Our study indicates that *T. bellirica* and *T. chebula* extracts are effective inhibitors of the growth of bacterial pathogens that cause gastrointestinal infections. This indicates that these extracts may have distinct antibacterial mechanisms that necessitate further investigation. Furthermore, all extracts improved the efficacy of several reference clinical antibiotics, particularly β-lactam antibiotics and tetracycline. It is possible that antibiotic reactivation may be occurring in these bacterial strains, which are otherwise resistant to the effects of these antibiotics. The potentiation mechanism remains to be determined, although specific extract components may inhibit bacterial β-lactamase enzymes (including extended spectrum enzymes) and/or efflux pumps, resulting in elevated antibiotic concentrations within the cells. Further research is necessary to validate these mechanisms and to explore additional possible enhancement pathways. Several phytochemicals identified in the *T. bellirica* and *T. chebula* fruit extracts may contribute to these activities, indicating that they could be promising targets for the development of novel antibacterial agents, although additional research is required. An assessment of the activities of the pure phytochemicals and their abilities to enhance conventional antibiotic activities should be the topic of future study.

## Figures and Tables

**Table 1:** MIC values (µg/mL) for the *T. bellirica* and *T. chebula* aqueous, methanolic, and ethyl acetate extracts, as well as the positive control antibiotics, against the bacterial species tested in this study.

Extract Type or Antibiotic	Bacterial Species and MIC (µg/mL)			
	*B. cereus*	*S. flexneri*	*S. sonnei*	*S. typhimurium*
TB-AQ	392 ± 0	392 ± 0	1569 ± 0	**3138 ± 0**
TB-MeOH	94 ± 0	377 ± 0	755 ± 0	755 ± 0
TB-EtOAc	450 ± 0	450 ± 0	>10,000	>10,000
TCh-AQ	556 ± 0	278 ± 0	**2225 ± 0**	**2225 ± 0**
TCh-MeOH	377 ± 0	377 ± 0	1509 ± 0	**3019 ± 0**
TCh-EtOAc	306 ± 0	306 ± 0	1225 ± 0	>10,000
PENG	**0.625 ± 0**	>2.5	>2.5	>2.5
ERY	**0.08 ± 0**	>2.5	>2.5	>2.5
TET	**0.08 ± 0**	**0.625 ± 0**	**0.31 ± 0**	**0.625 ± 0**
CHL	**1.25 ± 0**	**1.25 ± 0**	**2.5 ± 0**	**2.5 ± 0**
CIP	**0.08 ± 0**	**0.02 ± 0**	**0.02 ± 0**	**0.02 ± 0**
POL	>2.5	**0.31 ± 0**	**0.31 ± 0**	**0.31 ± 0**
OXA	**1.25 ± 0**	>2.5	>2.5	>2.5
AMX	**0.625 ± 0**	>2.5	>2.5	>2.5
GEN	**0.16 ± 0**	**0.625 ± 0**	**2.5 ± 0**	**0.625 ± 0**
VAN	**0.625 ± 0**	>2.5	>2.5	>2.5

All samples and controls were tested in two independent experiments, each with internal duplicates (n = 4), and are expressed as mean MIC values ± SEM. MIC values are categorised as inactive (MIC > 10,000 µg/mL), low activity (**2000–5000** µg/mL; indicated in bold), moderate activity (1000–2000 µg/mL; indicated in blue), noteworthy activity (400–1000 µg/mL; indicated in red), good activity (100–400 µg/mL; indicated in green), or potent activity (<100 µg/mL; highlighted in yellow). TB-AQ = *T. bellirica* aqueous; TB-MeOH = *T. bellirica* methanolic; TB-EtOAc = *T. bellirica* ethyl acetate. TCh-AQ = *T. chebula* aqueous; TCh-MeOH = *T. chebula* methanol; TCh-EtOAc = *T. chebula* ethyl acetate. The antibiotics included PENG (penicillin G), ERY (erythromycin), TET (tetracycline), CHL (chloramphenicol), CIP (ciprofloxacin), POLB (polymyxin B), OXA (oxacillin), AMX (amoxycillin), VAN (vancomycin), and GEN (gentamicin). Values for active antibiotics are shown in bold, while those lacking antibiotic activity in this study possessed MICs greater than 2.5 µg/mL.

**Table 2:** ∑FIC values determined for the relevant combinations of *T. bellirica* or *T. chebula* extracts with conventional antibiotics.

Bacteria	Extract	PENG	ERY	TET	CHL	CIP	POLB	OXA	AMX	GEN	VAN
***B. cereus***	TB-AQ	0.56	2.00	1.00	1.06	2.00	-	1.03	0.56	1.50	0.56
	TB-MeOH	1.03	1.25	1.25	1.02	1.25	-	1.01	1.03	1.13	2.07
	TB-EtOAc	1.50	2.12	2.12	2.00	1.06	-	1.50	1.50	8.81	1.50
	TCh-AQ	0.56	1.50	1.50	1.13	1.50	-	0.53	0.81	2.00	1.26
	TCh-MeOH	0.78	2.00	1.00	0.53	2.00	-	0.52	0.56	0.75	1.13
	TCh-EtOAc	0.51	2.25	2.25	1.50	2.25	-	0.62	1.00	2.44	1.50
***S. flexneri***	TB-AQ	-	-	1.13	1.06	2.00	5.00	-	-	0.56	-
	TB-MeOH	-	-	1.13	1.06	1.50	5.00	-	-	1.13	-
	TB-EtOAc	-	-	0.75	1.00	1.06	15.75	-	-	3.00	-
	TCh-AQ	-	-	1.13	1.06	1.50	5.00	-	-	1.13	-
	TCh-MeOH	-	-	1.13	1.06	2.00	5.00	-	-	1.13	-
	TCh-EtOAc	-	-	1.00	1.50	2.25	16.00	-	-	4.00	-
***S. sonnei***	TB-AQ	-	-	1.00	0.56	1.25	8.50	-	-	1.12	-
	TB-MeOH	-	-	0.75	1.06	3.00	9.00	-	-	0.53	-
	TB-EtOAc	-	-	3.00	3.00	3.00	4	-	-	4	-
	TCh-AQ	-	-	0.75	0.62	1.12	32.25	-	-	0.62	-
	TCh-MeOH	-	-	1.00	1.12	2.50	16.50	-	-	0.56	-
	TCh-EtOAc	-	-	1.12	1.00	2.06	63.00	-	-	4	-
***S. typhimurium***	TB-AQ	-	-	1.00	1.25	2.44	3.00	-	-	1.50	-
	TB-MeOH	-	-	0.75	1.12	2.00	2.00	-	-	0.75	-
	TCh-AQ	-	-	1.00	1.25	2.44	6.03	-	-	2.00	-
	TCh-MeOH	-	-	1.00	1.25	2.44	3.00	-	-	2.00	-

*T. bellirica* and *T. chebula* aqueous extracts (TB-AQ, TCh-AQ), methanolic extracts (TB-MeOH, TCh-MeOH), and ethyl acetate extracts (TB-EtOAc, TCh-EtOAc) were included. Additive interactions are represented by ∑FIC values of >0.5 to ≤1.00 (blue color values); indifferent interactions by >1.01 to ≤4.00 (black color values); and antagonistic interactions at values >4.0 (red color values). A dash (-) indicates that the combination could not be tested since an MIC could not be determined for either the extract or the antibiotic. TB-AQ = *T. bellirica* aqueous; TB-MeOH = *T. bellirica* methanolic; TB-EtOAc = *T. bellirica* ethyl acetate. TCh-AQ = *T. chebula* aqueous; TCh-MeOH = *T. chebula* methanol; TCh-EtOAc = *T. chebula* ethyl acetate. PENG = penicillin G, ERY = erythromycin, TET = tetracycline, CHL = chloramphenicol, CIP = ciprofloxacin, POL B = polymyxin B, OXA = oxacillin, AMX = amoxycillin, GEN = gentamicin, and VAN = vancomycin.

**Table 3:** The putative identification and % relative abundance of phytochemicals identified using LC-MS in the aqueous (Aq), methanolic (MeOH), and ethyl acetate (EtOAc) fruit extracts of *Terminalia bellirica* (TB) and *Terminalia chebula* (TCh).

*Retention Time (min)*	*Molecular Weight*	*Empirical Formula*	*Putative Compounds*	*% Relative Abundance (TB)*			*% Relative Abundance (TCh)*		
				*Aq*	*MeOH*	*EtOAc*	*Aq*	*MeOH*	*EtOAc*
*1.496*	*192.06305*	*C_7_H_12_O_6_*	*Quinic acid*	*3.13*	*0.33*	*0.19*	*0.39*	*-*	*1.53*
*1.573*	*344.07409*	*C_14_H_16_O_10_*	*Theogallin*	*-*	*3.47*	*-*	*-*	*0.03*	*0.02*
*1.784*	*302.06328*	*C_12_H_14_O_9_*	*Pyrogallol-2-O-glucuronide*	*-*	*0.21*	*-*	*0.07*	*-*	*-*
*2.045*	*294.03749*	*C_13_H_10_O_8_*	*Banksiamarin B*	*-*	*-*	*0.15*	*0.17*	*0.05*	*0.13*
*2.058*	*174.05268*	*C_7_H_10_O_5_*	*Shikimic acid*	*-*	*0.41*	*0.67*	*2.64*	*3.14*	*4.80*
*2.237*	*154.02646*	*C_7_H_6_O_4_*	*Gentisic acid*	*0.02*	*0.05*	*-*	*0.01*	*-*	*0.09*
*2.296*	*448.15769*	*C_19_H_28_O_12_*	*8-O-Acetyl shanzhiside methyl ester*	*-*	*-*	*-*	*0.19*	*0.18*	*-*
*2.356*	*332.07411*	*C_13_H_16_O_10_*	*6-Galloylglucose*	*6.06*	*3.97*	*3.39*	*3.74*	*0.68*	*-*
*2.444*	*448.06386*	*C_20_H_16_O_12_*	*Ellagic acid 2-rhamnoside*	*0.11*	*1.44*	*0.69*	*0.01*	*0.21*	*0.33*
*2.51*	*292.02177*	*C_13_H_8_O_8_*	*Brevifolincarboxylic acid*	*0.09*	*-*	*-*	*0.11*	*0.03*	*0.61*
*2.56*	*288.0844*	*C_12_H_16_O_8_*	*Phlorin*	*-*	*0.02*	*-*	*0.03*	*0.02*	*-*
*3.11*	*484.0848*	*C_20_H_20_O_14_*	*Gallic acid 3-O-(6-galloylglucoside)*	*0.74*	*-*	*-*	*-*	*-*	*-*
*3.158*	*212.06824*	*C_10_H_12_O_5_*	*Propyl gallate*	*-*	*-*	*-*	*0.30*	*0.35*	*0.12*
*4.4*	*277.05821*	*C_13_H_11_NO_6_*	*Salfredin C1*	*-*	*-*	*-*	*0.14*	*-*	*0.03*
*4.847*	*184.03703*	*C_8_H_8_O_5_*	*Methyl gallate*	*0.25*	*11.39*	*-*	*0.04*	*1.05*	*-*
*8.44*	*484.0855*	*C_20_H_20_O_14_*	*1,6-Bis-O-(3,4,5-trihydroxybenzoyl) hexopyranose*	*5.13*	*5.19*	*2.52*	*6.52*	*2.16*	*3.78*
*9.316*	*470.01176*	*C_21_H_10_O_13_*	*Sanguisorbic acid dilactone*	*-*	*0.08*	*-*	*0.56*	*0.61*	*0.19*
*9.567*	*634.08082*	*C_27_H_22_O_18_*	*Sanguiin H4*	*0.84*	*-*	*0.07*	*-*	*5.63*	*0.54*
*9.811*	*478.07452*	*C_21_H_18_O_13_*	*Miquelianin*	*0.04*	*0.42*	*-*	*0.37*	*0.60*	*0.39*
*9.902*	*152.04703*	*C_8_H_8_O_3_*	*Vanillin*	*-*	*-*	*-*	*0.16*	*0.17*	*0.17*
*9.921*	*126.03146*	*C_6_H_6_O_3_*	*Phloroglucinol*	*0.10*	*1.93*	*-*	*0.07*	*0.24*	*0.27*
*10.043*	*636.09614*	*C_27_H_24_O_18_*	*1,2,6-Trigalloyl-β-D-glucopyranose*	*0.09*	*5.44*	*2.85*	*0.11*	*1.42*	*2.22*
*10.419*	*484.08522*	*C_20_H_20_O_14_*	*Hamamelitannin*	*2.10*	*0.02*	*0.62*	*0.02*	*0.11*	*-*
*10.421*	*126.0316*	*C_6_H_6_O_3_*	*Pyrogallol*	*6.28*	*5.28*	*2.19*	*6.29*	*3.82*	*4.87*
*10.422*	*296.05248*	*C_13_H_12_O_8_*	*cis-Coutaric acid*	*0.33*	*0.86*	*0.19*	*0.31*	*0.93*	*1.05*
*10.55*	*601.99658*	*C_28_H_10_O_16_*	*Diellagilactone*	*-*	*-*	*-*	*1.13*	*0.60*	*-*
*10.703*	*372.1055*	*C_16_H_20_O_10_*	*Veranisatin C*	*0.02*	*0.04*	*-*	*-*	*-*	*-*
*10.754*	*636.09608*	*C_27_H_24_O_18_*	*1,3,6-Tri-O-galloyl-β-D-glucose*	*-*	*0.25*	*0.93*	*0.64*	*-*	*-*
*11.305*	*170.02138*	*C_7_H_6_O_5_*	*Phloroglucinic acid*	*0.98*	*2.80*	*4.33*	*0.98*	*0.83*	*1.24*
*11.091*	*610.1535*	*C_27_H_30_O_16_*	*Rutin*	*-*	*0.14*	*0.10*	*-*	*0.03*	*0.08*
*11.176*	*432.10539*	*C_21_H_20_O_10_*	*Vitexin*	*-*	*-*	*-*	*-*	*0.05*	*-*
*11.448*	*302.04213*	*C_15_H_10_O_7_*	*Quercetin*	*0.11*	*0.33*	*0.17*	*0.02*	*-*	*-*
*11.473*	*464.0951*	*C_21_H_20_O_12_*	*Myricitrin*	*0.05*	*0.13*	*0.19*	*-*	*-*	*-*
*11.589*	*170.02147*	*C_7_H_6_O_5_*	*Gallic acid*	*26.23*	*8.64*	*2.60*	*23.90*	*1.90*	*1.56*
*11.822*	*462.07961*	*C_21_H_18_O_12_*	*Aureusidin 6-glucuronide*	*-*	*-*	*2.52*	*-*	*-*	*0.04*
*11.905*	*286.04729*	*C_15_H_10_O_6_*	*Maritimetin*	*-*	*-*	*-*	*T*	*0.01*	*0.01*
*12.183*	*448.0996*	*C_21_H_20_O_11_*	*Maritimein*	*-*	*0.01*	*0.02*	*-*	*-*	*-*
*12.185*	*302.00627*	*C_14_H_6_O_8_*	*Ellagic acid*	*2.45*	*10.74*	*33.49*	*8.39*	*10.46*	*11.96*
*12.233*	*262.0476*	*C_13_H_10_O_6_*	*Maclurin*	*-*	*0.02*	*-*	*0.04*	*-*	*-*
*12.254*	*148.05223*	*C_9_H_8_O_2_*	*trans-Cinnamic acid*	*-*	*-*	*-*	*0.76*	*0.96*	*0.87*
*12.503*	*192.07847*	*C_11_H_12_O_3_*	*(R)-Shinanolone*	*-*	*-*	*-*	*0.04*	*0.03*	*0.05*
*12.509*	*610.1894*	*C_28_H_34_O_15_*	*Neohesperidin*	*-*	*-*	*0.16*	*-*	*-*	*-*
*12.584*	*498.1741*	*C_23_H_30_O_12_*	*Eucaglobulin*	*-*	*0.02*	*-*	*-*	*-*	*-*
*12.593*	*436.13688*	*C_21_H_24_O_10_*	*Nothofagin*	*-*	*-*	*-*	*-*	*-*	*0.12*
*12.721*	*680.37692*	*C_36_H_56_O_12_*	*Tenuifolin*	*-*	*-*	*-*	*-*	*0.11*	*0.37*
*12.8*	*486.33397*	*C_30_H_46_O_5_*	*Bassic acid*	*-*	*-*	*-*	*-*	*-*	*0.05*
*12.844*	*518.1785*	*C_26_H_30_O_11_*	*Phellodensin E*	*-*	*-*	*0.05*	*-*	*-*	*-*
*12.889*	*346.1052*	*C_18_H_18_O_7_*	*Hamilcone*	*-*	*-*	*-*	*0.02*	*-*	*0.01*
*12.914*	*190.13544*	*C_13_H_18_O*	*β-Damascenone*	*-*	*-*	*-*	*-*	*0.02*	*0.06*
*12.915*	*666.39739*	*C_36_H_58_O_11_*	*Chebuloside II*	*-*	*-*	*-*	*-*	*3.85*	*-*
*12.924*	*302.1152*	*C_17_H_18_O_5_*	*Lusianin*	*0.01*	*-*	*-*	*-*	*-*	*-*
*12.929*	*356.03763*	*C_14_H_12_O_11_*	*(+)-Chebulic acid*	*0.54*	*-*	*-*	*3.98*	*1.40*	*2.88*
*12.969*	*504.34417*	*C_30_H_48_O_6_*	*Madecassic acid*	*-*	*-*	*-*	*-*	*-*	*1.42*
*12.971*	*202.13522*	*C_14_H_18_O*	*(±)-Anisoxide*	*-*	*-*	*-*	*-*	*-*	*0.05*
*12.973*	*244.14583*	*C_16_H_20_O_2_*	*Lahorenoic acid C*	*-*	*-*	*-*	*-*	*-*	*0.02*
*13.208*	*288.06318*	*C_15_H_12_O_6_*	*Eriodictyol*	*-*	*-*	*-*	*-*	*-*	*0.27*
*13.428*	*442.1992*	*C_25_H_30_O_7_*	*Exiguaflavanone M*	*-*	*-*	*0.13*	*-*	*-*	*-*
*13.451*	*274.08371*	*C_15_H_14_O_5_*	*Phloretin*	*-*	*-*	*-*	*0.01*	*0.02*	*0.01*
*13.536*	*462.11641*	*C_22_H_22_O_11_*	*Leptosin*	*-*	*0.01*	*-*	*0.01*	*0.06*	*-*
*13.59*	*302.04255*	*C_15_H_10_O_7_*	*Bracteatin*	*-*	*-*	*-*	*0.01*	*0.01*	*-*
*13.593*	*502.1836*	*C_26_H_30_O_10_*	*Flavaprin*	*-*	*-*	*0.07*	*-*	*-*	*-*
*13.678*	*568.12178*	*C_28_H_24_O_13_*	*Isoorientin 2″-p-hydroxybenzoate*	*-*	*-*	*-*	*-*	*-*	*0.04*
*13.703*	*280.13067*	*C_15_H_20_O_5_*	*Artabsinolide A*	*-*	*-*	*-*	*-*	*-*	*0.02*
*13.805*	*444.10499*	*C_22_H_20_O_10_*	*3′-O-Methylderhamnosylmaysin*	*-*	*-*	*-*	*0.04*	*0.12*	*0.08*
*13.805*	*292.09447*	*C_15_H_16_O_6_*	*(S)-Angelicain*	*-*	*-*	*-*	*0.05*	*-*	*-*
*14.024*	*500.1674*	*C_26_H_28_O_10_*	*Ikarisoside A*	*-*	*-*	*0.03*	*-*	*-*	*-*
*14.078*	*534.28285*	*C_29_H_42_O_9_*	*Corchoroside A*	*-*	*-*	*-*	*-*	*-*	*0.02*
*14.358*	*272.06832*	*C_15_H_12_O_5_*	*Naringenin*	*-*	*-*	*-*	*-*	*-*	*0.18*
*14.367*	*470.33907*	*C_30_H_46_O_4_*	*Glycyrrhetinic acid*	*-*	*-*	*-*	*-*	*0.08*	*0.01*
*14.783*	*234.16167*	*C_15_H_22_O_2_*	*Valerenic acid*	*-*	*-*	*-*	*-*	*-*	*0.13*
*14.928*	*202.17175*	*C_15_H_22_*	*Rulepidadiene B*	*-*	*-*	*-*	*-*	*-*	*0.08*
*14.93*	*222.16153*	*C_14_H_22_O_2_*	*Rishitin*	*-*	*-*	*-*	*-*	*-*	*0.04*
*15.046*	*426.09477*	*C_22_H_18_O_9_*	*Epiafzelechin 3-O-gallate*	*-*	*-*	*-*	*-*	*-*	*0.01*
*15.454*	*650.4026*	*C_36_H_58_O_10_*	*Pedunculoside*	*-*	*-*	*-*	*-*	*1.18*	*2.61*
*16.257*	*738.41926*	*C_39_H_62_O_13_*	*Isonuatigenin 3-[rhamnosyl-(1->2)-glucoside]*	*-*	*-*	*-*	*-*	*0.04*	*0.12*
*16.301*	*470.1941*	*C_26_H_30_O_8_*	*Limonin*	*-*	*-*	*0.14*	*-*	*-*	*-*
*16.478*	*540.1663*	*C_25_H_32_O_11_S*	*Sumalarin B*	*-*	*-*	*0.02*	*-*	*-*	*-*
*16.532*	*316.1308*	*C_18_H_20_O_5_*	*Methylodoratol*	*-*	*-*	*0.02*	*-*	*-*	*-*
*16.787*	*472.2097*	*C_26_H_32_O_8_*	*Kushenol H*	*-*	*-*	*0.07*	*-*	*-*	*-*
*16.844*	*344.0532*	*C_17_H_12_O_8_*	*3,4,3′-Tri-O-methylellagic acid*	*-*	*-*	*0.01*	*-*	*-*	*-*
*16.876*	*252.20856*	*C_16_H_28_O_2_*	*Isoambrettolide*	*-*	*-*	*-*	*-*	*-*	*0.32*
*17.064*	*440.1828*	*C_25_H_28_O_7_*	*Lonchocarpol E*	*-*	*-*	*0.15*	*-*	*-*	*-*
*17.209*	*544.2668*	*C_30_H_40_O_9_*	*Physagulin F*	*-*	*-*	*0.10*	*-*	*-*	*-*
*17.276*	*180.11471*	*C_11_H_16_O_2_*	*Jasmolone*	*-*	*0.61*	*1.48*	*0.05*	*-*	*-*
*17.31*	*504.34409*	*C_30_H_48_O_6_*	*Protobassic acid*	*-*	*-*	*-*	*1.26*	*0.02*	*0.01*
*17.33*	*468.32326*	*C_30_H_44_O_4_*	*Glabrolide*	*-*	*-*	*-*	*0.87*	*-*	*3.19*
*17.33*	*502.32964*	*C_30_H_46_O_6_*	*Medicagenic acid*	*-*	*-*	*-*	*-*	*T*	*0.07*
*17.331*	*200.15605*	*C_15_H_20_*	*(S)-gamma-Calacorene*	*-*	*-*	*-*	*0.02*	*-*	*0.29*
*17.363*	*696.40877*	*C_37_H_60_O_12_*	*Momordicoside E*	*-*	*-*	*-*	*-*	*0.01*	*0.04*
*17.626*	*286.08357*	*C_16_H_14_O_5_*	*Homobutein*	*-*	*T*	*0.01*	*-*	*-*	*0.01*
*17.635*	*282.12541*	*C_18_H_18_O_3_*	*Ohobanin*	*0.01*	*T*	*0.09*	*T*	*-*	*0.06*
*17.671*	*652.27302*	*C_32_H_44_O_14_*	*Dicrocin*	*-*	*-*	*-*	*-*	*-*	*0.03*
*17.902*	*424.1881*	*C_25_H_28_O_6_*	*Paratocarpin G*	*T*	*-*	*-*	*-*	*-*	*-*
*17.949*	*456.2144*	*C_26_H_32_O_7_*	*Antiarone J*	*-*	*0.54*	*-*	*-*	*-*	*-*
*18.124*	*372.2509*	*C_20_H_36_O_6_*	*Sterebin Q4*	*-*	*-*	*0.01*	*-*	*-*	*-*
*18.237*	*428.1831*	*C_24_H_28_O_7_*	*Heteroflavanone B*	*-*	*-*	*0.05*	*-*	*-*	*-*
*18.237*	*312.13615*	*C_19_H_20_O_4_*	*Desmosdumotin C*	*T*	*-*	*0.02*	*T*	*-*	*0.02*
*18.292*	*546.35535*	*C_32_H_50_O_7_*	*Hovenidulcigenin B*	*-*	*-*	*-*	*0.04*	*-*	*-*
*18.339*	*236.1776*	*C_15_H_24_O_2_*	*Capsidiol*	*-*	*-*	*-*	*-*	*-*	*0.02*
*18.346*	*268.13069*	*C_14_H_20_O_5_*	*Kamahine C*	*0.02*	*0.01*	*-*	*-*	*0.01*	*0.15*
*18.363*	*336.0994*	*C_20_H_16_O_5_*	*Ciliatin A*	*-*	*-*	*-*	*-*	*0.01*	*-*
*18.498*	*340.09447*	*C_19_H_16_O_6_*	*Ambanol*	*-*	*-*	*-*	*-*	*0.01*	*-*
*18.507*	*484.31803*	*C_30_H_44_O_5_*	*Liquoric acid*	*-*	*-*	*-*	*T*	*0.01*	*0.04*
*18.623*	*488.35017*	*C_30_H_48_O_5_*	*Pitheduloside I*	*-*	*-*	*-*	*T*	*0.06*	*0.11*
*19.641*	*226.09903*	*C_15_H_14_O_2_*	*7-Hydroxyflavan*	*-*	*0.84*	*-*	*0.03*	*-*	*0.05*
*18.748*	*208.1096*	*C_12_H_16_O_3_*	*Isoelemicin*	*0.01*	*-*	*0.19*	*-*	*-*	*-*
*18.767*	*526.2565*	*C_30_H_38_O_8_*	*Kosamol A*	*-*	*0.52*	*-*	*-*	*-*	*-*
*18.987*	*372.1208*	*C_20_H_20_O_7_*	*Tangeretin*	*-*	*-*	*0.03*	*-*	*-*	*-*

% Relative abundance is the ratio of a single compound’s signal intensity to the total signal intensity of all detected compounds in a chromatogram. The percentage represents the compound’s contribution to the overall chromatographic profile. However, the percentage relative abundance does not represent the actual quantity of the compound in the sample; rather, it shows its proportion based on signal strength, which might vary depending on the detector’s response to different compounds. The % abundance data were obtained from a single, representative chromatogram for each extract. Compounds identified in both plants extracts are in black; compounds identified only in *Terminalia bellirica* (TB) extracts are in red; compounds identified only in *Terminalia chebula* (TCh) extracts are in green; compounds less than 0.01% of the total area were considered as trace amounts and denoted as T.

## Data Availability

Data are either presented within the manuscript or are available from the corresponding author upon reasonable request.
